# Hyper-realistic face masks: a new challenge in person identification

**DOI:** 10.1186/s41235-017-0079-y

**Published:** 2017-10-25

**Authors:** Jet Gabrielle Sanders, Yoshiyuki Ueda, Kazusa Minemoto, Eilidh Noyes, Sakiko Yoshikawa, Rob Jenkins

**Affiliations:** 1 Department of Psychology, University of York, Heslington, York, YO10 5DD UK; 2 Kokoro Research Center, Kyoto University, Kyoto, Japan

**Keywords:** Masks, Silicone, Realistic, Face perception, Face recognition, Passports, Identification, Uncanny valley, Deception, Fraud

## Abstract

We often identify people using face images. This is true in occupational settings such as passport control as well as in everyday social environments. Mapping between images and identities assumes that facial appearance is stable within certain bounds. For example, a person’s apparent age, gender and ethnicity change slowly, if at all. It also assumes that deliberate changes beyond these bounds (i.e., disguises) would be easy to spot. Hyper-realistic face masks overturn these assumptions by allowing the wearer to look like an entirely different person. If unnoticed, these masks break the link between facial appearance and personal identity, with clear implications for applied face recognition. However, to date, no one has assessed the realism of these masks, or specified conditions under which they may be accepted as real faces. Herein, we examined incidental detection of unexpected but attended hyper-realistic masks in both photographic and live presentations. Experiment 1 (UK; n = 60) revealed no evidence for overt detection of hyper-realistic masks among real face photos, and little evidence of covert detection. Experiment 2 (Japan; n = 60) extended these findings to different masks, mask-wearers and participant pools. In Experiment 3 (UK and Japan; n = 407), passers-by failed to notice that a live confederate was wearing a hyper-realistic mask and showed limited evidence of covert detection, even at close viewing distance (5 vs. 20 m). Across all of these studies, viewers accepted hyper-realistic masks as real faces. Specific countermeasures will be required if detection rates are to be improved.

**Electronic supplementary material:**

The online version of this article (doi:10.1186/s41235-017-0079-y) contains supplementary material, which is available to authorized users.

## Significance

In several high-profile criminal cases, offenders have used hyper-realistic face masks to transform their appearance, leading police to pursue suspects who look nothing like the offenders themselves (e.g., different race or age). In other settings, airline passengers wearing hyper-realistic masks have boarded international flights without the deception being noticed. Such incidents are likely to become more common as hyper-realistic masks become easier to manufacture. These developments have potentially far-reaching implications for security and crime prevention. Face identification requires a one-to-one mapping between faces and people, so that appearance can be traced to identity unambiguously. If viewers do not distinguish between hyper-realistic masks and real faces, the mapping can be compromised, and facial appearance is no longer informative for identification. We find that viewers fail to detect hyper-realistic masks, even when they attend to facial appearance. Exceptions to this pattern hint at possible methods for improving detection performance.

## Background

Face recognition is a common means of identifying people and an important component of security and crime prevention internationally. For example, passport issuance (White, Kemp, Jenkins, Matheson, & Burton, 2014) and passport control (McCaffery & Burton, 2016) both involve facial image comparison. Conviction of criminal suspects can sometimes hinge on eyewitness testimony (Wells & Olson, 2003; Bruce, 1988; https://www.innocenceproject.org) or CCTV footage (Burton, Wilson, Cowan, & Bruce, 1999; Davis & Valentine, 2009). In many countries, a photo-ID is required for the purchase of age-restricted goods (Gosselt, van Hoof, de Jong, & Prinsen, 2007; Vestlund, Langeborg, Sörqvist, & Eriksson, 2009). Because face identification carries such weight in these situations, it is also a major focus for identity fraud and deception (Robertson, Kramer, & Burton, 2017). In particular, individuals may wish to impersonate someone else or to avoid being recognised themselves (Dhamecha, Singh, Vatsa, & Kumar, 2014).

**Fig. 1:**
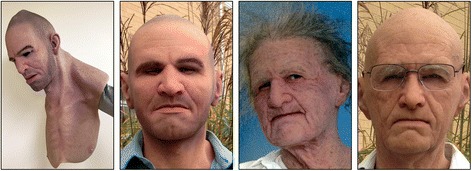
Hyper-realistic silicone masks. Images show (from left to right) a young male mask (YMM), followed by a young male mask (YMM), an old female mask (OFM) and an old male mask (OMM) worn by author RJ

One way to conceal identity is simply to cover the face, for example, using fabric or a mask (Fecher & Watt, 2013). Covering the face is generally effective in obscuring identity (Burton et al., 1999), but it is also visually and socially salient, and likely to arouse the suspicion of onlookers (Zajonc, 1968). Over the past decade, this limitation has been challenged by the emergence of hyper-realistic, hand-painted silicone masks (Fig. 1), originally developed in the special effects industry as an alternative to multi-hour make-up sessions. The flexibility and strength of silicone confer several advantages in this situation. Unlike traditional masks that cover the face only, a silicone mask may cover the whole head and neck so that it extends below the collar without any joins. This seamless construction creates the impression that the visible face is part of a continuous body surface rather than being a separate overlay (Anderson, Singh, & Fleming, 2002). Realism is further enhanced by transmission of non-rigid movement (e.g., rotation of the head relative to the body, opening and closing of the mouth, gross changes in facial expression) from the surface of the face to the surface of the mask. Importantly, the wearer’s real eyes, nostrils and mouth cavity are all visible through the mask via close-fitting holes that match the topology of the face beneath. Several manufacturers offer hand-punched human hair and stubble as optional extras.

These advances in mask fabrication raise the question of how realistic a mask can be. For the present purposes, we adopt a pragmatic definition of realism, namely a mask is realistic if it is perceived as a real face. This criterion has the advantage of being testable and can be applied across different viewers and viewing conditions. It also gets to the heart of the practical problem. If covering one’s face arouses suspicion, the ability to cover one’s face without arousing suspicion would seem to favour the deceiver.

There are reasons to doubt that this level of realism can be achieved in practice. For one, the visual system is highly attuned to face stimuli, including subtleties of skin tone (Fink, Grammer, & Matts, 2006; Frost, 1988; Bindemann & Burton, 2009) and face shape (Oosterhof & Todorov, 2008; Ekman, 2003). Thus, it seems plausible that even minor departures from authentic appearance at the physical level could loom large at the perceptual level. Paradoxically, some demands of the perceptual system may become harder to satisfy as authenticity increases. The ‘uncanny valley’ refers to the phenomenon whereby human response to humanoid artifacts (e.g., robots, dolls, puppets) shifts from empathy to revulsion as the humanoid approaches, but fails to attain, lifelike appearance (Mori, 1970; see Mori, MacDorman, & Kageki, 2012, for an English language translation). Given humans’ particular sensitivity to face stimuli, one might expect the uncanny valley to pose a particular challenge for masks (Seyama & Nagayama, 2007). A sense of eeriness could undermine an otherwise compelling overall impression of realism.

Theoretical concerns aside, the important question is whether these masks actually fool anyone. There is now a good deal of anecdotal evidence that hyper-realistic masks can pass for real faces in everyday life. In one incident, a white bank robber used a silicone mask to disguise himself as a black man for a string of robberies in the USA. Six out of seven bank tellers wrongly identified a black man as the culprit in a photo line-up; only when the robber’s girlfriend intervened was the black suspect released from jail (Bernstein, 2010). In another case, a young Asian man disguised himself as an elderly white man using a silicone mask and boarded a flight from Hong Kong to Canada (Zamost, 2010). The deception was only detected when the passenger removed the mask midflight and a fellow traveller brought the change in appearance to the attention of the crew. These examples imply that realistic masks can be mistaken for real faces, even when the viewer’s attention is focused on facial appearance (as is the case in police line-ups and passport checks). Surprisingly, however, there has been no experimental research into hyper-realistic masks and the conditions under which they can be detected.

Herein, we address these questions in three experiments. We examine mask detection from static photographs (Experiment 1 and 2) and in live viewing (Experiment 3) to assess performance in these two modes of face identification. We had the opportunity to collect data from both British and Japanese participants, allowing us to compare performance for own-race and other-race faces. A large body of research on the other-race effect has shown that identification performance is more reliable for own-race faces than for other-race faces (Meissner & Brigham, 2001). Our question here is whether a similar bias operates when distinguishing hyper-realistic masks from real faces.

## Experiment 1

In Experiment 1, we secretly embedded photos of hyper-realistic masks among photos of real faces. Participants worked through these photos sequentially, rating the person in each photo on a series of social dimensions. This task ensured that participants processed the images, but did not draw attention to the distinction between real faces and masks. We then asked a series of graded questions to determine whether or not they had noticed any masks among the faces. After explaining the manipulation, we showed the stimuli again and asked participants to pick out any photos that contained masks. We predicted that, when participants were not expecting to see masks (i.e., during the rating phase), realistic masks might not be detected, resulting in few spontaneous reports of masks in post-test questioning. However, when participants are expecting to see masks (i.e., after the manipulation has been explained), they should be able to distinguish realistic masks from real faces, merely by inspecting the photographs.

### Method

#### Ethics statement

Ethical approval was granted by the departmental ethics committee at the University of York.

#### Participants

Sixty undergraduate and postgraduate members of the volunteer panel at the University of York (10 males; mean age = 21, age range 18–39 years) took part in exchange for a small payment or course credit.

#### Stimuli and design

We used three different mask models from Realflesh Masks, Quebec, Canada – *The Pensioner* (Old Male Mask), *The Fighter* (Young Male Mask) and *The Grandma* (Old Female Mask). The company offers a range of hair options for its masks. We opted for punched human hair eyebrows on all three and a full head of hair on *The Grandma*.

To generate mask images, we took multiple photographs of the same volunteer model wearing each of the three masks. We took photos indoors and outdoors under different viewing conditions to approximate the range of variability seen in natural face images (Jenkins, White, Van Montfort, & Burton, 2011). For each mask, we selected two different photos that depicted the mask in frontal view with no occlusions (six mask images in total).

To generate real face images, we entered the terms ‘young male’, ‘old male’, ‘young female’ and ‘old female’ into Google Image search. For each of these four face types, we selected the first five colour photos of unfamiliar Caucasian faces that (1) exceeded 200 pixels in height, (2) showed the face in roughly frontal aspect and (3) were free from occlusions (20 real face images in total). All photos (masks and real faces) were cropped to show the head region only and resized to 540 pixels high × 385 pixels wide for presentation.

Starting with the 20 real face photos, we created different stimulus sets by substituting one mask for one real face of the same type (young male, old male, or old female). This resulted in six variant image sets, each consisting of one mask photo embedded in 19 real face photos. Ten participants saw each variant.

#### Procedure

**Fig. 2:**
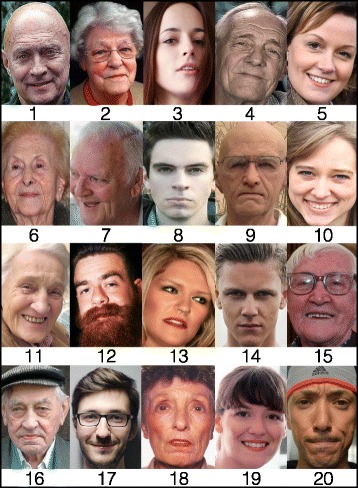
Example array challenge from Experiment 1. Participants were asked to indicate any photos that show a mask. The array always contained 19 real face photos and 1 mask photo. In this example, image 9 shows author RJ in the old male mask (OMM)

Participants viewed 20 photographs (19 real faces and 1 hyper-realistic mask), one at a time, in a random order. To encourage deep processing of facial appearance, we asked participants to estimate the age of the person in each photo, and to rate the person for ‘Trustworthiness’, ‘Dominance’ and ‘Attractiveness’, using a 7-point Likert scale. There was no time limit for this task and photos remained on screen until all responses were made. This rating task was followed by a series of graded questions to assess detection of the mask. Question 1, ‘What did you think of the faces you saw?’, was deliberately open and was intended to capture spontaneous, overt detection of the mask. Question 2, ‘Did you notice anything unusual about any of the faces?’, encouraged participants to report any suspicions that they may have had during the task (i.e., more covert detection). Both of these questions invited typed responses. Question 3, ‘In this experiment, half of the participants are in the *Mask* group (where at least one of the photos contains a mask). The other half are in the *No Mask* group (where none of the photos contained a mask). Which group do you think you were in (*Mask* vs. *No Mask*)?’ led to a two-alternative forced choice (2AFC), which was intended to provide a more sensitive measure. After responding, participants were informed that they were in the *Mask* group. They were then presented with all 20 of the photos they had rated (19 real faces and 1 mask) in a randomly ordered 5 × 4 array and asked to indicate any photo that contains a mask (Question 4; Fig. 2). At the end of the experiment, participants were debriefed and asked to indicate whether or not they had prior knowledge of realistic silicone masks before the start of the experiment.

### Results

#### Mask detection

**Fig. 3:**
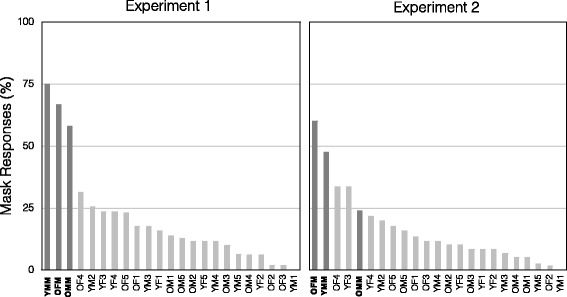
Responses to the array challenge in Experiment 1 (left) and Experiment 2 (right). Bars show, for each image in the array, the percentage of participants who reported it as a mask, and are ordered by frequency. Dark bars represent mask images (*YMM* young male mask, *OFM* old female mask, *OMM* old male mask). Light bars represent real face images (*YM* young male, *OM* old male, *YF* young female, *OF* old female)

We first tested for overt detection of the masks by analysing the content of typed responses to Question 1 (‘What did you think of the faces you saw?’) and Question 2 (‘Did you notice anything unusual about any of the faces?’). To avoid imposing our own interpretations on these responses, we simply coded for the presence (1) or absence (0) of the word ‘mask’ in the text. As it turned out, none of the 60 participants included the word ‘mask’ in either response. That is, there were no cases of overt detection (see Additional file 1 for raw data). For the 2AFC item (Question 3), only 21.7% of participants guessed that they were in the *Mask* group, significantly lower than the chance level of 50% (t(59) = 5.28, *P* < 0.001, *d* = −17). Finally, in the array challenge (Question 4), 70% of participants correctly picked out the mask. However, participants also picked out an average of 2.5 (range 0–10) real faces (Fig. 3, left). In fact, all but one of the real faces (YM1) was reported as a mask at least once. χ^2^ analysis revealed no significant differences in detection performance across mask types (2AFC: χ^2^ (3, n = 60) = 0.79, *P* = 0.68, Cramer’s *v* = 0.13; Array challenge: χ^2^ (3, n = 60) = 1.43, *P* = 0.490, *v* = 0.12).

#### Mask knowledge

Overall, 38 of the 60 participants declared prior knowledge of hyper-realistic masks. χ^2^ analyses revealed no significant difference in 2AFC performance between *Knowledge* (n = 38; 21.1%) and *No Knowledge* (n = 22; 22.7%) subgroups (χ^2^ (2, n = 60) = 0.02, *P* = 0.807, *v* = 0.03). However, prior knowledge conferred a significant advantage in the array challenge (*Knowledge*: 78.9%; *No Knowledge*: 54.5%; χ^2^ (3, n = 60) = 3.95, *P* = 0.046, *v* = 0.28).

### Discussion

We find it quite striking that not a single participant volunteered that they had seen a mask. Even under 2AFC questioning, only 22% thought that a mask might have been presented. These findings suggest that, at least in the context of viewing photos, participants need to both (1) be informed that a mask may be present and (2) have the images available for inspection, if they are to distinguish hyper-realistic masks from real faces. Even when these conditions were met (in the array challenge), 30% of participants missed the mask and 78% picked out at least one real face. The message from this experiment is that detecting hyper-realistic masks is hard, even when the test conditions are highly favourable. We next consider a situation in which the test conditions may be less favourable – viewing other-race faces.

## Experiment 2

Viewers are generally poor at identifying other-race faces compared with own-race faces. This is true for tasks involving recognition memory (Meissner & Brigham, 2001) and also for tasks involving perceptual comparison of face photographs (e.g., Megreya, White, & Burton, 2011). The perceptual explanation of this own-race bias is that the ability to distinguish individuals is refined by experience, namely that viewers become attuned to the variability that surrounds them and remain relatively insensitive to variability outside of this range (O’Toole, Deffenbacher, Valentin, & Abdi, 1994). This differential sensitivity supports finer perceptual discriminations for own-race faces than for other-race faces. In the case of hyper-realistic masks, distinguishing a mask from a real face also requires fine perceptual discriminations, perhaps akin to distinguishing one person from another. If so, the task of hyper-realistic mask detection may also be susceptible to own-race bias. In Experiment 2, we had the opportunity to replicate Experiment 1 in Japan, using the same stimuli and procedure as before, but now with Japanese participants. Given that all of our stimuli showed Western (Caucasian) faces and masks, our main interest was whether hyper-realistic masks would be more readily accepted by Japanese participants compared with the UK participants in Experiment 1.

### Method

#### Ethics statement

Ethical approval was obtained from the Kokoro Research Center ethics committee at Kyoto University.

#### Participants

Sixty undergraduate and postgraduate members of the volunteer panel at Kyoto University (36 males; mean age = 22, age range 19–36 years) took part in exchange for a small payment.

#### Stimuli and procedure

The stimuli, design and procedure were exactly as for Experiment 1, except that the task instructions were now translated into Japanese. Two experienced translators provided translations independently. The best translation was selected and verified for functional similarity with the English version by a third, bilingual English-Japanese speaker.

### Results

#### Mask detection

Consistent with Experiment 1, none of the 60 participants mentioned the Japanese word for ‘mask’ in response to Question 1 or Question 2 (see Additional file 2 for raw data). For the 2AFC item (Question 3), 33.3% of participants guessed that they were in the *Mask* group, significantly below chance (t(59) = 2.72, *P* = 0.009, *d* = −10). Finally, in the array challenge (Question 4), just 45% of participants correctly picked out the mask. Participants picked out an average of 2.3 (range 0–11) real faces (Fig. 3, right). As in Experiment 1, all but one of the real faces (YM1) was identified as a mask at least once. Again, there were no significant differences in detection performance across mask types (2AFC: χ^2^ (3, n = 60) = 2.17, *P* = 0.338, *v* = 0.103; Array challenge: χ^2^ (3, n = 60) = 3.75, *P* = 0.074, *v* = 0.27).

#### Mask knowledge

Only three participants in the Japanese sample reported prior knowledge of hyper-realistic masks. Of the 57 participants who had no prior knowledge of masks, 32.2% guessed that they were in the mask group (Question 3) and 47% picked the mask in the array challenge (Question 4). Of the three participants who reported prior knowledge, one picked the mask group (Question 3) and two picked the mask out of the array correctly (Question 4).

#### Comparison of UK and Japan samples

None of the 120 participants (60 UK, 60 Japan) mentioned masks spontaneously (Question 1) or when prompted (Question 2). For the 2AFC item (Question 3), the proportion of ‘mask’ responses was higher for Japanese participants (33.3%) than for UK participants (21.7%), though this difference was not significant (χ^2^ (1, n = 120) = 2.05, *P* = 0.152, v = 0.14). However, in the array challenge (Question 4), Japanese participants picked out the actual mask significantly less often (46.7%) than the UK participants (70%) (χ^2^ (1, n = 120) = 6.72, *P* = 0.010, *v* = 0.24).

### Discussion

Overall, the results are very similar to those seen in Experiment 1. Like the UK viewers, Japanese viewers did not spontaneously report seeing a mask despite two opportunities to do so (Questions 1 and 2). A low proportion of viewers believed that they were in the *Mask* condition (Question 3) and a low proportion picked the mask out from an array of real face photos (Question 4). Accuracy on this array challenge was reliably lower in Experiment 2 (Japanese viewers) than in Experiment 1 (UK viewers), possibly reflecting an other-race effect, although there are many other possible explanations for this difference.

To follow on from these findings, we expanded to a fully crossed design in which both British and Japanese participants viewed both Asian and Western faces. More importantly, we also progressed from viewing photographs on a computer screen to viewing live faces outdoors.

## Experiment 3

Mask detection rates in the preceding experiments were consistently low. There are several reasons to be cautious in interpreting this finding. One is that all of the stimuli in Experiments 1 and 2 were photographic images. Single, static photos present much less information than dynamic, live faces (Jenkins & Burton, 2011). It is possible that, under live viewing conditions, detection rates could be much higher. On the other hand, all of the participants knew that they were taking part in a psychology experiment, and this setting may have made them especially vigilant. On that basis, it is possible that under live viewing conditions, detection rates could be even lower.

To avoid these limitations, we adapted the mask detection measures from Experiments 1 and 2 to a very different situation. Instead of recruiting participants to a laboratory-based experiment, we recruited passers-by in an outdoor area of the University. Additionally, instead of asking these volunteers to rate onscreen photographs, we asked them about a live confederate. In one condition, the confederate wore a hyper-realistic mask. As in the previous experiments, our main interest was whether viewers noticed the mask or accepted it as a real face (*High-realism mask* condition). To establish a false alarm rate, we included a condition in which the confederate did not wear a mask (*Real face* condition). To establish the rate of miss errors (due to inattention, misunderstanding task instructions, etc.), we also included a condition in which the confederate wore a highly salient party mask (*Low-realism mask* condition). This allowed us to assess the detection rate for hyper-realistic masks relative to these base-rates.

To test for other-race effects in this task, we recruited participants in both Japan and the UK to view both Asian and Western masks. An other-race effect should result in poorer detection of hyper-realistic masks for *Other-race* trials (Japanese participants viewing Western masks and British participants viewing Asian masks), compared with *Own-race* trials (Japanese participants viewing Asian masks and British participants viewing Western masks). Finally, we examined effects of viewing distance by comparing performance in *Near* (5 m) and *Far* (20 m) conditions. We expected improved detection of high-realism masks at the closer viewing distance, where more detail is visible.

### Method

#### Ethics statement

Ethical approval was granted by the Kokoro Research Center ethics committee at Kyoto University and the departmental ethics committee at the University of York.

#### Participants

A total of 407 volunteers participated in the study. All participants were undergraduate or postgraduate students at the University of York, UK (n = 199; 107 males; mean age = 20, age range 18–44 years) or Kyoto University, Japan (n = 208; 134 males; mean age = 21 years, age range 18–38 years).

#### Stimuli and design

**Fig. 4:**
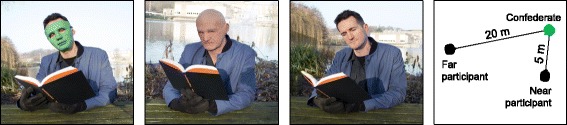
Illustration showing (from left to right) author RJ in the *Low-realism mask*, *High-realism mask* and *Real face* conditions of Experiment 3, and the spatial arrangement of confederate and participants

Four male confederates were briefed on the aims of the study. For the *High-realism mask* condition, we used four masks in total. Three of these were produced by Realflesh Masks, Quebec, Canada – *The Pensioner* (Western old male mask), *The Fighter* (Western young male mask) and *The Asian* (Asian old male mask). The remaining mask was the *Jae* model (Asian young male mask), by Composite Effects (CFX), Los Angeles, USA. We ordered punched human hair eyebrows on all four masks, a goatee beard and horseshoe hair on *The Asian* and a full head of hair on the *Jae*. To avoid overcomplicating the design, confederates wore own-race masks only. For the *Low-realism mask* condition, we used two visually salient masks that covered the face only, rather than the whole head. These were a plain green Halloween-style mask (Fig. 4) and a black butterfly-shaped masquerade mask. Note that the distinction between *Own-race* and *Other-race* applies to the *High-realism mask* condition and the *Real face* condition, but does not apply to the *Low-realism mask* condition.

Combining each of these presentations with *Near* and *Far* viewing distances resulted in 10 conditions in total. Each participant saw one condition only (between-subjects design). As in the preceding experiments, each participant responded to an open question, a prompted question and a 2AFC question.

#### Procedure

Testing took place in campus courtyards at the University of York and Kyoto University between 11:00 and 14:00 on different dry weather days between November 2014 and October 2016. For the duration of the testing session, the confederate remained seated at a bench in a university courtyard with reliable foot traffic. The two experimenters recruited viewers at approximately 5 m (*Near* condition) and 20 m (*Far* condition; Fig. 4, right panel) from the confederate by pointing out the confederate to individual passers-by and asking whether they would mind answering a few questions about him. To encourage deep processing of facial appearance, the participant was first asked to rate the confederate for ‘Trustworthiness’, ‘Dominance’ and ‘Attractiveness’, using a 7-point Likert scale. After responding, the participant was asked to turn to the experimenter so that the confederate was no longer in view. The experimenter then asked graded mask detection questions that were adapted from the preceding experiments: ‘What did you think of that person?’ (Open question), ‘Did you notice anything unusual about the person?’ (Prompted question) and ‘There are two conditions in this experiment, one where the person is wearing a mask and one where he is not wearing a mask. Which condition are you in?’ (2AFC question). Data were recorded by the experimenters using prepared response sheets. The entire procedure lasted approximately 2 minutes for each participant.

### Results

#### Descriptives

Table 1 summarizes the distribution of participants across conditions.

**Table 1:** Number of participants tested in each of the 10 different conditions in Experiment 3, shown separately for testing in UK and Japan. Note that the Own-race / Other-race distinction does not apply to the Low-realism mask condition

Test Location	Viewing distance	Low-realism mask	High-realism mask		Real face	
			Own-race	Other-race	Own-race	Other-race
Japan	Near (5 m)	24	20	20	20	20
	Far (20 m)	23	20	20	20	21
UK	Near (5 m)	20	20	22	20	20
	Far (20 m)	18	20	18	20	21

#### Mask detection

**Fig. 5:**
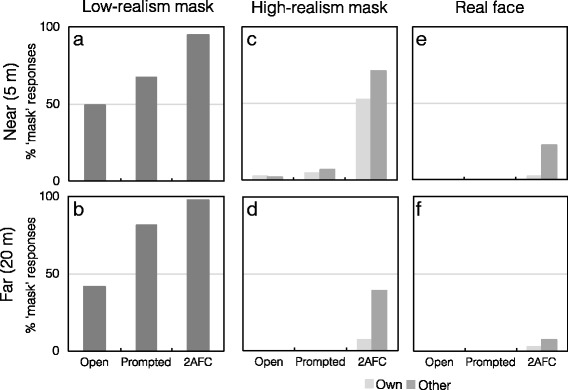
Mask detection data from Experiment 3. Bars show the percentage of ‘mask’ responses to Open, Prompted and 2AFC questions about the experimental confederate. Responses are broken down by realism (**a**, **b** *Low-realism mask*; **c**, **d** *High-realism mask*; **e**, **f** *Real face*) and by viewing distance (**a**, **c**, **e** *Near*; **b**, **d**, **f** *Far*). For the *High-realism mask* and *Real face* conditions, responses are shown separately for *Own-race* (light grey) and *Other-race* (mid grey). Sample sizes for each panel: **a**, 44; **b**, 41; **c**, 82; **d**, 78; **e**, 81; **f**, 81

To ensure consistency across experiments, we coded responses to Questions 1 and 2 according to the presence or absence of the word ‘mask’ in the response. As expected, detection rates in the *Low-realism mask* group were high overall (Fig. 5), indicating good engagement with the task. For the Open question (Question 1), 49.2% of *Near* participants and 42.1% of *Far* participants included the word ‘mask’ in their responses. For the Prompted question (Question 2), these proportions rose to 67.2% (*Near*) and 82.3% (*Far*). Finally, for the 2AFC item (Question 3), almost all participants guessed that they were in the *Mask* group (*Near* 95.0%, *Far* 97.9%). In sum, *Low-realism masks* were rarely missed.

Complementing this pattern, performance in the *Real face* group shows a low false alarm rate. None of the participants in this group used the word ‘mask’ in their responses to either the Open question (Question 1) or the Prompted question (Question 2). For the 2AFC item (Question 3), participants in the *Own-race* condition almost never guessed that they were in the ‘mask’ group (*Near* 2.5%, *Far* 2.5%). Interestingly, participants in the *Other-race* group occasionally picked the ‘mask’ group, especially those at the closer viewing distance (*Near* 22.5%, *Far* 7.5%). This observation may be important for interpreting the pattern of results. For now, the main message is that real faces were rarely mistaken for masks.

The critical issue is the performance of the *High-realism mask* group relative to the two comparison groups. Of the 160 participants in this group, only two (1.3%) used the word ‘mask’ in their responses to the Open question. For the Prompted question, this number rose to five (3.1%). All five of these participants were in the *Near* condition. Given this very low rate of spontaneous detection, the rest of the analysis focuses on responses to the 2AFC item (Question 3).

### Analysis of 2AFC responses

#### Effects of realism

Figure 5 shows a clear separation between the *Low-realism mask* and *Real face* conditions, with intermediate performance in the *High-realism mask* condition. χ^2^ analysis confirmed a significant difference between conditions (χ^2^(1) = 179.28, *P* < 0.001, *v* = 0.66). Post-hoc tests revealed that ‘mask’ responses in the *High-realism* condition (42.5%) were significantly less frequent than in the *Low-realism* condition (96.5%; χ^2^(1) = 141.61, *P* < 0.001, *v* = 0.53) and significantly more frequent than in the *Real face* condition (8.6%; χ^2^(1) = 112.61, *P* < 0.001, *v* = 0.39). Interestingly, the rate of ‘mask’ responses in the *High-realism* condition was not significantly different from 50% (χ^2^(1) = 3.60, *P* = 0.058), indicating low consensus or low confidence in these responses.

#### Effects of race

The rate of ‘mask’ responses was higher overall in the *Other-race* condition than in the *Own-race* condition (χ^2^(1) = 16.23, *P* < 0.001, *v* = 0.22). Importantly, this effect was present not only in the *High-realism* condition (χ^2^(1) = 12.38, *P* < 0.001, *v* = 0.28), but also in the *Real face* condition (χ^2^(1) = 7.55 *P* = 0.005, *v* = 0.22), suggesting that it may reflect a decision bias rather than a difference in perceptual discrimination.

#### Effects of viewing distance

Overall, ‘mask’ responses were more frequent in the *Near* condition than in the *Far* condition (χ^2^(1) = 16.66, *P* < 0.001, *v* = 0.21). Post-hoc comparisons revealed that this effect was due to increased mask responses in the *High-realism* condition only (χ^2^(1) = 26.70, *P* < 0.001, *v* = 0.41). There was no effect of viewing distance for the *Real face* condition (χ^2^(1) = 2.66, *P* = 0.103, *v* = 0.13) or the *Low-realism* condition (χ^2^(1) = 0.42, *P* = 0.52, *v* = 0.07).

### Discussion

Hyper-realistic masks were very rarely detected in this experiment. At the longer viewing distance (20 m), no one in the *High-realism* condition reported a mask. Even at close range (5 m), only 2 out of 82 viewers reported a mask spontaneously, rising to 5 out of 82 for the prompted question. For the 2AFC question, the proportion of participants who guessed that they were in the *Mask* condition ranged from 7.5% (*Own-race*, *Far* condition) to 71% (*Other-race*, *Near* condition), depending on race and viewing distance. Importantly, these factors similarly affected responses in the *Real face* condition.

One possible explanation for the elevated ‘mask’ responses in the *Other-race* condition is that participants’ judgements incorporated demographic base-rates. In Japan, Western faces are less frequent than Asian faces. In the UK, Asian faces are less frequent than Western faces. This uneven distribution gives rise to different prior probabilities. At the same time, the finding that ‘mask’ responses were more frequent in the *High-realism mask* condition than in the *Real face* condition, and more frequent in the *Near* condition than in the *Far* condition, implies that subtle visual cues also played a role. Taken together, these observations suggest separable contributions from prior probability and visual evidence to participants’ decisions.

## General discussion

Part of our interest in hyper-realistic masks stems from their use in security settings. At first sight, it is difficult to credit that a person wearing a full mask could board a plane unchallenged. How are we to make sense of such incidents? Do they reflect inattention on the part of the observer, or perhaps an unwillingness to confront the mask wearer? Or could it be that, in these situations, hyper-realistic masks are indistinguishable from real faces? In our experiments, almost no one reported noticing the mask, despite attending to the mask and answering several questions about its appearance. This was true for photographic images presented onscreen. It was also true for live confederates presented outdoors. The numbers are sobering. Of the 280 participants who viewed hyper-realistic masks in these studies (60 in Experiment 1; 60 in Experiment 2; 160 in Experiment 3), only two spontaneously reported the mask and only three more reported the mask following further prompting. Interestingly, all five of these participants viewed the mask live (Experiment 3) and at the closer viewing distance of 5 m. These are low detection rates. Evidently, the information available even in near-distance, live viewing (visual detail, 3D form, motion) did not allow viewers to distinguish hyper-realistic masks from real faces with any generality. Nevertheless, the clustering of these few participants by viewing condition suggests that the available information may have some diagnostic value, above and beyond that which is available at longer viewing distances or in photographic presentations.

Other aspects of our results bear out this interpretation. In Question 3 of each experiment, we asked participants to guess whether they were in the *Mask* condition or the *No Mask* condition (2AFC). The intention here was to draw out more covert detection of hyper-realistic masks, perhaps arising from an uncanny valley phenomenon. We anticipated that the wording of Question 3, combined with the sensitivity of 2AFC as a measure, might lead to a ceiling effect in responses, with all participants guessing that they were in the *Mask* condition. As it turned out, 2AFC performance did not approach ceiling in any of the experiments (with the planned exception of the low-realism masks in Experiment 3). Instead, ‘mask’ responses were the minority in Experiment 1, Experiment 2 and the *Far* condition of Experiment 3. Even in the *Near* condition of Experiment 3, ‘mask’ responses were not reliably above 50%.

Presumably, there must be some critical distance at which viewers spontaneously and accurately distinguish hyper-realistic masks from real faces. After all, painted silicone and human skin are different materials with different surface properties (Motoyoshi, Nishida, Sharan, & Adelson, 2007). We do not know what this critical distance might be, but we can now be confident that the *Near* distance in Experiment 3 (5 m) exceeds it. That finding may have implications for mask detection in the real world. Classic work on proxemics (Hall, 1966) divides interpersonal space into four radial zones. In this scheme, intimate distance (0–1.5 feet; 0–0.5 m) is associated with physical contact and whispering, personal distance (1.5–4 feet; 0.5–1.2 m) is reserved for interactions among close friends or family, social distance (4–12 feet; 1.2–3.7 m) accommodates interactions among acquaintances, and public distance (>12 feet; > 3.7 m) is occupied by strangers. Our upper bound of 5 m suggests that any critical distance for mask detection falls within social space (4–12 feet; 1.2–3.7 m) or closer in this scheme. Nevertheless, most people do not enter this space. Strangers in particular tend to be seen at longer range, where we now know mask detection is unreliable. One important exception is photo-ID checks (e.g., passport control), which are typically carried out at a distance of one or two metres (Verhoff, Witzel, Kreutz, & Ramsthaler, 2008; Noyes & Jenkins, 2017). Future studies should assess mask detection performance at this closer range. However, anecdotal reports of mask use on airlines (Zamost, 2010) and the prevalence of identification errors in live-to-photo comparisons (Kemp, Towell, & Pike, 1997; Davis & Valentine, 2009; White et al., 2014) do not inspire confidence.

These proxemic considerations raise some interesting questions about the appearances of hyper-realistic masks and their social effects. To date, mask manufacturers have followed a single strategy for evading detection, namely the pursuit of ever greater realism. An interesting direction for future research would be to assess the viability of a complementary strategy: evading detection by manipulating the behaviour of onlookers. It is almost tautological that the less approachable a mask looks, the less inclined viewers will be to approach it and the less likely they will be to reach the critical distance for detection. A similar argument could be made for attractiveness. To the extent that facial attractiveness summons attention (Shimojo, Simion, Shimojo, & Scheier, 2003; Sui & Liu, 2009) and increases dwell time (Leder, Tinio, Fuchs, & Bohrn, 2010), a less attractive mask should receive less scrutiny. Based on such principles, it may be possible to devise a hyper-realistic mask that deflects observers’ minds by (1) maximising viewing distance and (2) minimising visual attention. A brutish-looking pickpocket might arrive at a different set of priorities, favouring a highly approachable mask that allows them to move closer to a target.

In future studies, it would be interesting to isolate the information that leads viewers to guess that they are in the *Mask* condition. The fact that ‘mask’ responses were more prevalent in the *Near* condition than the *Far* condition suggests that high spatial frequency information plays an important role. However, it is not clear whether decisions are driven by local visual features (e.g., surface discontinuities around the eyes or mouth), by more holistic visual features (e.g., wrinkle patterns over the whole face), or by higher-level inferences that are abstracted from such information (e.g., social attributions based on facial appearance). If reliable cues can be established, they could potentially form the basis of a training program aimed at enhancing mask detection. For passive viewing situations, such as reviewing recorded footage, this could be as simple as encouraging observers to monitor for particular visual features.

For interactive situations, such as live identity checks, more active approaches may be feasible. Our informal observation is that wearing a hyper-realistic mask attenuates some forms of facial movement. Even with good contact between the face and the mask, manipulating the mask places additional demands on facial muscles, relative to normal facial movement. Moreover, movements that may be clear and distinct at the internal surface of the mask (where they are initiated) will be partly absorbed by the silicone on their way to the external surface (where they are seen). These attenuation effects may be negligible for coarse movements such as rotation of the head on the neck, and opening and closing of the jaw. Nevertheless, emotional expressions such as smiles and frowns generally appear muted, and subtle expressions are often lost altogether.

The overall facial impression, at least in extended interactions, is one of blunted animacy. It is possible that, under appropriate testing conditions, this impression might be enough to cue detection of a hyper-realistic mask, perhaps by tipping the interaction into the uncanny valley. However, it may also encourage false positives for low-animacy real faces. Thus, blunted animacy in the face may be more diagnostic when it is paired with incongruous animacy cues from the body or voice. Various aspects of facial appearance, including apparent age, gender and emotion, can shape viewers’ expectations about how a person is likely to move and speak (e.g., Lander, Hill, Kamachi, & Vatikiotis-Bateson, 2007; Johnson, McKay, & Pollick, 2011; Van den Stock, Righart, & De Gelder, 2007; Montepare & Zebrowitz-McArthur, 1988). Violations of those expectations, such as sprinting centenarians, may allow viewers to infer the presence of a mask, even if the mask itself is entirely convincing.

Speech could be revealing for other reasons too. Normal speech comprehension is strongly supported by visual lip-reading (Campbell, 2008; McGurk & MacDonald, 1976). However, the lips of a hyper-realistic mask fully cover the lips of the wearer (Fig. 1). This arrangement has a number of implications for speech and lip-reading. First, it introduces a physical barrier between the wearer’s lips, presumably impeding production of phonemes that require contact between the lips (e.g.,/b/,/p/,/m/), or between the teeth and the lower lip (e.g.,/f/,/v/). Second, it reduces the pliability of the whole mouth area, presumably impeding articulation more generally. Reduced lip movement implies reduced visual support for speech understanding (Campbell, 2008). It also suggests that hyper-realistic masks may affect the auditory stream in distinctive ways. Ironically, auditory information may provide the best hope of solving this difficult visual task.

Perception of emotional expression, uncanny valley effects, cue integration and speech comprehension are all matters that can be unpicked experimentally. Our observation (Experiment 1) of elevated detection rates for participants with prior knowledge of hyper-realistic masks suggests that training to enhance performance is possible at least in principle. The optimal form of training remains to be determined.

We also tested for other-race effects in mask detection. Other-race effects were originally observed in the context of face identification – a task that requires fine perceptual discriminations. Given that distinguishing hyper-realistic masks from real faces also requires fine perceptual discriminations, we wondered whether performance would be poorer for other-race faces than for own-race faces. The evidence on this particular point was not very clear. Floor effects in the Open question and Prompted question make it difficult to draw any conclusions about race effects in overt detection, beyond noting that the task defeated own-race and other-race viewers alike. The same manipulation did have some impact on responses to the 2AFC item, but even here the different experiments present a mixed picture. Experiment 1 (UK participants) and Experiment 2 (Japanese participants) were both based entirely on Western face images. Comparing across experiments, Japanese viewers were somewhat more likely than UK participants to guess that they were in the *Mask* condition (rather than the *No Mask* condition), but this difference was not statistically significant. Experiment 3, using a fully crossed design and a larger sample, found a significant difference in the same direction, namely that other-race viewers were more likely than own-race viewers to guess that they were in the *Mask* condition. On its own, this effect might suggest an other-race advantage in distinguishing real faces from hyper-realistic masks, which would contrast with the other-race disadvantage that is standard in identification tasks. However, the *Real face* condition undermines this interpretation – for real faces, too, other-race viewers were disproportionately likely to guess that they were in the *Mask* condition. That finding is not consistent with an other-race advantage in distinguishing real faces from hyper-realistic masks. Instead, it suggests an overall bias towards guessing ‘mask’.

This interpretation of the 2AFC data accords with the array challenge findings (Experiments 1 and 2). In the array challenge, Japanese participants picked out the mask significantly less often than the UK participants. Given that the stimuli were Western face images, this pattern resembles the expected disadvantage for other-race faces. It is not obvious how one might square an other-race disadvantage in the array challenge with an other-race advantage in the 2AFC. However, no such tension arises between an other-race disadvantage in the array challenge and a decision bias in the 2AFC.

Why might other-race viewers be especially inclined to guess that they are in the *Mask* condition? One possibility is that, at least in the campus locations tested, other-race faces are simply less prevalent than own-race faces. That being the case, if the confederate presents an other-race face, the participant has to explain the balance of probabilities. Either they just happen to be witnessing a (relatively) rare event, or they are subject to an experimental manipulation. Presumably, some proportion of participants finds the latter explanation more compelling than the former. If this argument is sound, we expect that equating the frequencies of own-race and other-race stimuli in a laboratory experiment should give rise to an other-race disadvantage.

Hyper-realistic masks fool most people most of the time. This finding should be unsettling, not least because it indicates a new frontier in deception. Covering the face may be grounds for suspicion when the intent is to conceal identity. Yet, historically, such deception has been easy to detect. In hyper-realistic masks, we confront the prospect of face coverings that shroud the wearer, yet are themselves accepted as real faces. It is difficult to estimate how many of these masks are already in circulation. However, as documented cases attest, their proliferation poses a challenge for face recognition in applied settings, including crime prevention and border control. We expect that increasingly sophisticated manufacturing techniques will continue to improve the quality of these masks and to drive prices down. Keeping pace with these improvements will require increasingly sophisticated countermeasures, perhaps including consciousness raising, personnel development and supplementary imaging methods. Machine vision researchers have made some interesting progress on this front (e.g., Erdogmus & Marcel, 2014; Kose & Dugelay, 2013). The conditions are conducive to a new arms race in face identification between deception and detection.

## Additional files

Additional file 1: Responses to open and prompted questions in Experiment 1. (DOCX 137 kb)

Additional file 2: Responses to open and prompted questions in Experiment 2. Translated from Japanese (original response in brackets). (DOCX 33 kb)

## Abbreviations

2AFC: two-alternative forced choice
