# Supplementary material for: Hyper-realistic face masks: a new challenge in person identification
Source: Cogn Res Princ Implic. 2017 Oct 25;2:43. doi: 10.1186/s41235-017-0079-y (PMC5655619; doi:10.1186/s41235-017-0079-y)
Supplement: Supplementary file 1 — Responses to open and prompted questions in Experiment 1. (DOCX 137 kb) [file 41235_2017_79_MOESM1_ESM.docx]

**Supplementary Materials 1: Responses to open question (‘What do you think of the faces you saw?’) and prompted question (‘Did you notice anything unusual?’) in Experiment 1**

| **P.No** | **Open response** | **Prompted response** |
| --- | --- | --- |
| **2** | i am female so for me, when i saw female faces, i think they are more trustworthy. and male with normal or smiling faces are also trustworthy. and one' s eys can reveal whether they are trustworthy or attractive. | one, why there were no children faces. two, it is odd that one female had freckles in her face, but i can see she had done some makeup. third, two of the old men looked very similar but just different facial expressions, one smiled one didn' t, which the former of looked trustworthy and attractive and the latter one reversely. |
| **3** | They all had similar expressions | No |
| **4** | Some of them were fairly easy to rate, facial expressions weighted some of my judgement. I have noticed that there was more older people picture displayed than that of younger people. | Some of the pictures looked like the colouring has been changed or was just a bit different to normal when the photo was taken. I didn' t notice any particular case where I would find a face unusual |
| **5** | There was a wide range of people to rate regarding their attractiveness and age. | No |
| **6** | people between 50 to 80 seem to be more trustwprthy. those who are smiling seem to be more attractive and trustworthy. people with a cold face seem to be dominant. | some faces with too many freckles and wrinkles may affect judgment directly. |
| **7** | they all looked like typical members of the public you would encounter on the street | quite a few of them were older |
| **8** | the faces seemed relatively trustworthy, i didn' t find any of the faces | no |
| **9** | they were a range of ages, gendars and ethnicities. Smiling ones looked friendlier so more trustworthy. hard to guess their age | Angry ones tended to be on a darker background |
| **10** | i did not notice anything unusual |  |
| **11** | The majority were quite elderly so I rated their looks mostly according to their eyes which tend to be the most attractive features in elderly people. I tend to find people who are smiling naturally or behaving calmly to be trustworthy so I rated faces wish such attributes accordingly. I did not see much in the way of diversity when looking at the faces and I tend to find European faces generally less attractive than other ethnicities. | Many were elderly which I was not expecting in an experiment asking us to rate attraction. I saw little in the way of ethnic diversities. |
| **12** | they were very varied and i found it quite hard to judge the older peoples ages | There was one face that I thought looked about mid 30' s and was bald, his face didnt look fully human, it had a plastic aspect to it |
| **13** | those people have different facial expressions which may influence my judgement about their age or attractiveness. most of the faces are young people or senior people, the middle aged are rare. overall, those faces are ordinary. | there is a girl with dimple on her face which is unsual to me |
| **14** | most photos made the person come across as very friendly and kind, therefore most faces seemed trustworthy | no |
| **15** | A lot of them were quite old and with some it was difficult to decide because they were pulling faces | Not really, they all looked quite normal |
| **16** | they were very different from one another | there were a lot more older faces |
| **17** | i thought one of them was a young person in old people' s make up | yes, i noticed that one of the images of a bald man was probably a younger man dressed in make up so that he looks older. i typed his age as 40 although the make up suggested he was older |
| **18** | varied expressions, mostly young adults or elderly, no middleaged faces. varied in approachable look due to smiling or stern expressions. most seemed to be happy, smiling or content | one face obscured by shadow, angle of faces varied e.g more stern looking tilted head upwards to narrow eyes. |
| **19** | found the more attractive faces to generally appear less dominant, and the older faces to appear more trustworthy | nothing unusual |
| **20** | They were diverse | Some people were making faces in them |
| **21** | older people are more trustworthy, young people are more attractive.people who have beautiful faces are more dominant. | a girl who has freckles is not attractive. |
| **22** | v | not really |
| **23** | A mix of many faces of moderate attraction, most seemed nice but some seemed less nice | Not really, other than some faces had odd expressions |
| **24** | a range of expressions from smiling and warm, to cold and glaring. | Only that some pictures were taken with flash, nothing about the faces themselves |
| **25** | The faces were composed of a variety of ages, gender and expressions, so the people smiling looked more friendly and therefore more trustworthy. | Some of the faces were in darker lighting so they looked more untrustworthy |
| **26** | They varied a lot | Some of them looked as though they were posing for a photo but a couple looked as though the photo was taken in the moment |
| **27** | not many faces are smiles | the texture of the skin, some faces have beautifual eyes |
| **28** | The faces seemed to either express happiness or anger. They seemed to be either young, middle aged or old. All faces were of one race. | One picture was very pixelated which made the face look like a drawing. |
| **29** | I thought that they were, on the most part, having their faces measured by the expression that they had on their face at the time of the photograph as this affects my perception of trustworthiness etc. | I did not notice anything particularly unusual about the faces themselves, no. |
| **30** | very wide range of faces from all different age groups, the hardest part was trying to work out their age particularly the elder participants. | one man had extremely small eye, unaware as to how this may have been caused. |
| **31** | they seemed to be either young or quite old | difference in age |
| **32** | most of them are just general public, some of them may be a bit of aggressive. | Some of the pictures are not good for ' judging', half of the face in shadow or in an angry expression or only three quarters of the whole. |
| **33** | I thought that the older people rated as more trustworthy and the younger people as more attractive but that was rather obvious | no not really |
| **34** | There was a very diverse and interesting collection of faces. | A couple of the faces were partially obscured by shadow. One girl had a vast amount of freckles on her face. |
| **35** | Most trusted either the older or kinder faces, faces that were either covered or at an angle were much harder to judge. Faces with glasses more trustworthy but only really shown on older people. | Faces with freckles was hard to give answers for other than that again covered or angled faces were hardest to judge |
| **36** | ranged in age nd gender, they varied in scale of trustworthy, attractive and dominant . elderly people seemed to look more trustworthy and men seemed more dominant | different expressions and different ages either young or old |
| **37** | They were very diverse, and interesting. Some faces were much harder to guess ages, eg older faces. I found that the younger faces seemed a lot more attective and most of the time more dominant than the older faces. | The only unusual things that I noticed were things like when people had no teeth, glasses or were a different ethinicity to previous faces. However i do remember noticing that one girl had a lot more frecles on her face. |
| **38** | different faces from different age groups | not really |
| **39** | smiling faces were generally more attractive, imperfections could be endearing and therefore make the face seem attractive, but sometimes work in the opposite way. a genuine smile also makes the person seem more trustworthy and less dominant | one man had very close together eyes, one woman had very freckly skin, one young man had quite bad acne, one of the older men had no teeth and one very pink shiny skin. one old lady had very wrinkly skin |
| **40** | they averaged from 20 to 80 years of age. they also varied with their attractiveness, dominion and trustworthy | no |
| **41** | Tend to rate older adults as more trustworthy. Hard to determine age of elderlies. | Some of the faces are not natural, e.g. photoshopped to change the brows, add more freckles, add more contrasts etc. One of the picture of a female elderly is highly altered e.g. added wrinkles, more contrasts, brightness and hues altered etc. |
| **42** | I think that it was difficult to judge the trustworthiness and dominance although age was ok. They are all different people. There weren' t many different ethnicities of people. | They weren' t all pulling the same face so this may have had an impact on my perception of their dominance and trustworthiness. The pictures were all tightly cropped to not allow anything other than their face impact my decision. |
| **43** | There was a wide range of faces varying in age, gender and ethnicity. I felt there was more old faces than younger faces. I felt also that my ratings depended on the facial expression they were displaying e.g. if the blonde woman was pulling a cross face she would have got a lower rating on trustworthiness than i gave her despite all her other features being the same. | Just that some of them were pulling faces. Also that one girl who had loads of freckles all over her face. I also felt that there was more older faces than younger faces. I felt that when they were older i was more likely to view them as trustworthy than if they were younger. |
| **44** | It was difficult to rate them without thinking about how they would look if they had different expressions on their faces. I tried to imagine them differently, especially for trustworthiness, which probably brought my answers more to the middle.I don' t remember seeing any noncaucasian faces. The older people made me smile more often, and I rated their attractiveness according to what I would think were I of the same age range. | Nothing unusual... |
| **45** | Mixed bag, no discernable characteristics on an aggregate level | Nope |
| **46** | the attractiveness of the face can affect the trustworthy of this person. the more the face looks nice, the easier the people trust he or she. old people with smile also are easier to be trusted. | one of them has lots of black spots on her face, it makes people feel scarey. it makes her less trustworthy. |
| **47** | they were mostly of older people and the faces showed emotion and personality to a small extent | some faces were especially fierce looking and intense |
| **48** | the most of the faces i saw are old people and they seems more trustworthy than young people. | most of the faces i saw are usual but one face with freckle is bit unusual because it is too much. |
| **49** | some of the faces are very attractive with a sunny smile, comfortable eye contact. faces that wear glasses and without smile seem to be more serious, dominant and trustworthy.both of the two kind of faces seem to be trustworthy.however, faces that carry strange expressions seem to be less reliable. | yes, some of the faces looks very comfortable while some faces wear expressions that look very sneaky. |
| **50** | Sample set seemed slightly skewed towards older ages. Lighting in one of the samples was obscured, potentially leading the viewer towards a certain response. It is difficult to ponder on the dominance and trustworthiness of others based on a snapshot alone. | See previous answer about obscurity. Also, one or two samples appeared as though they had suffered from some disease or other, melanoma and other cancers perhaps. As such, I tried to reduce my age prediction based on this likely making the sample appear older than usual. |
| **51** | a good mix of male and females, mostly smiling. Old people always seem sweeter and nicer and happier somehow. very few middle aged people though. Mostly young or old. That, or I cannot tell people' s age. | brighter photos tended to be happier and darker photos tended to be frowning or angry looking. Not sure whether this may have affected trustworthiness. |
| **52** | A large range of different faces, some of the expressions made me a bit uncomfortable so that I wanted to answer questions quicker. I noticed that there was a bigger range of ages than there was of races. | Some of the old people were quite scary, and people who were looking straight at the camera seemed more dominant. The less attractive people were less trustworthy. |
| **53** | They were very varied, most of them were trustworthy and there were a lot of elderly people. | There were a lot of older faces, and a few had a specific expression, whereas most were neutral or just similing |
| **54** | The elderly faces were more difficult to determine the age | no not really |
| **55** | the younger faces were easier to guess the age than older faces, facial expression on the pictures influenced my responses in all 3 areas | none of the young faces looked very happy, only the elderly people looked happy. Older faces were of similar ethinicity, younger faces were more diverse, with different facial expressions too |
| **56** | i thought they were all were different ages and that they all pulled similar expressions for the camera. | one picture of an old lady had a different sort of colouring to the others. |
| **57** | i may have responded differently to the faces that were pulling an aggressive expresion if they had a more neutral one. | no |
| **58** | They were all showing some sort of mood expression and had generally quite distinctive features. The majority seemed to be relatively old. | Some of the faces were what society would think were normal, and some were not. For example an elderly women with bad teeth and a younger person with freckles. However there was nothing particularly unusual which I could notice. |
| **59** | they seemed very generic faces all what you would imagine someone to look like given a descriotion none had unsual characteristics | no |
| **60** | All very different when it comes to mood, clothing, hair colour, but most of them look like nice persons, except from one which looked like a drawing. | One of the faces looked like a drawing, and looked like a criminal. |
| **61** | There was a wide range of faces, but it was hard to compare one' s trustworthiness to another for example because of different expressions and situations. | There seemed to be a few very posed faces which may influence people' s opinions of dominance etc. These contrasted a lot with the more nautral photos. Also the differing quality in the photos used. Nothing unusual directly about the faces per se. |
