# Supplementary material for: Hyper-realistic face masks: a new challenge in person identification
Source: Cogn Res Princ Implic. 2017 Oct 25;2:43. doi: 10.1186/s41235-017-0079-y (PMC5655619; doi:10.1186/s41235-017-0079-y)
Supplement: Supplementary file 2 — Responses to open and prompted questions in Experiment 2. Translated from Japanese (original response in brackets). (DOCX 33 kb) [file 41235_2017_79_MOESM2_ESM.docx]

**Supplementary Materials 2: Responses to open question (‘What do you think of the faces you saw?’) and prompted question (‘Did you notice anything unusual?’) in Experiment 2. Translated from Japanese (original response in brackets).**

| **P.No** | **Open response** | **Prompted response** |
| --- | --- | --- |
| **1** | Facial expression and atmosphere  (*hyoujou to huinki*) | Nothing  (*nanimo*) |
| **2** | Whether they smile or not  (*Egaokadouka*) | Nothing  (*Tokuninasi*) |
| **3** | I felt that they smiled a lot  (*Egaogaooinatokannjita*) | I haven’t noticed anything particularly (*Tokunihakidukanakatta*) |
| **4** | Nothing  (*Tokuninanimo*) | English written by participant: No |
| **5** | Age, whether they are dominant or not, and whether they are attractive or not.  (nenrei,shihaitekimiryokuteisinraidekirukadouka) |  |
| **6** | I think Eastern people looked younger than Western people did, so I was considering this when I guessed age  (*touyoukei no hito wa seiyoukei no hito yori wakaku mieru keikou ni aru to omou node sonokoto wo kouryo sinagara nennrei wo suitei sita*) | Nothing  (*toku ni nakatta to omou*) |
| **7** | Whether I have a good impression about her or him (*koukanwomoterujinbutsuka*) | I did not realise anything  (*kigatsukimasendesita*) |
| **8** | Whether there were face lines, or the size of the face. How sagging the face was. Facial expression. Or the sincerity of facial expressions. For age, I looked at face lines and how sagging the face was. For attractiveness, dominance and sincerity, I judged based on my sense of beauty and intuition  (*kao no siwa no umu, ookisa . kao no tarumi. hyoujou. mata hyoujou no seijitusa. watashiha nennreini tuiteha kaono siwa ya tarumi no katachi de, miryoku ya sihaido, seijitusa si tuite ha watasi jisinn no biteki kankaku ya chokkan de handanshimashita*) | Not at all. I did not realise anything  (*not at all. mattaku kidukimasendeshita)* |
| **9** | What kind of people the (presented) person was  (*donna hito nanoka*) | Some photos were processed  (*syashin ga kakou sareteiru mono ga aru)* |
| **10** | Impression at a glance  (pattomitekanjitainsyou) | Nothing  (tokuninasi) |
| **11** | The identity (something translated as: ‘true self’) of the person (*Sonojinnbutunosujyounado*) | I did not realise anything  (*Kidukanakatta*) |
| **12** | Older people looked less dominant than younger people did (*Toshiwototteiruhougashihaitekidehanasasou*) | Nothing  (*Tokuniarimasenn*) |
| **13** | Facial expressions and atmosphere  (*kao no hyoujou ya funiki*) | I did not notice anything at all  (*mattaku ki ga tsukanakatta*) |
| **14** | The first impression  (*Saisyonoinnsyou*) | Some people have a freckled face (*Sobakasugatakusannaruhitogaita*) |
| **15** | Age and occupation  (*nenrei to syokugyou*) | Nothing  (*Nashi*) |
| **16** | Whether I would trust this person or not. Something like this, I judged based on my criteria (*jibundattarakonohitowosinnraisurudarouka,nadoto,jibunwokijunnisitekangaemasita*) | I thought some photos were processed. (*mononiyotteha,kakousareteirunodehanaika,tokanjita.*) |
| **17** | Whether facial expressions were calm. I think I judged people’s impressions based on the sharpness (?) of eye stare (h*youjougaodayakasoukadouka,metukinosurudosadetaninnnoinnsyouwohanndannsiteiruyouniomou*) | There was no child photo  (*Kodomonosyasinganakatta*) |
| **18** | Facial expression, impression  (*hyouzyou innshou*) | There were two age populations (*Nennreisougahutatsuniwakareteiru*) |
| **19** | If the person were a teacher, I can trust or rely on what the person said etc.  (*moshi sonohitotachi ga sensei dattara to kateisite ,sonohito no iukoto wa shinnyoudekiruka toka izonshiteshimauka nado wo*) | Nothing  (*Tokuninashi*) |
| **20** | How I feel  (*Jibunngadoukanjitaka*) | There were a lot of weird facial expressions (*kimyounahyoujougaookatta*) |
| **21** | I looked at people’s eyes and judged when I evaluated each person (*Hitonohyoukawokudasutokimazumewomitekanngaeta*) | There were only Western faces and no Asian ones (*Seioujinnnokaobakarideajiakeinokawoganakatta*) |
| **22** | Mainly, I judged with wrinkles.  (omonishiwawomitehandanshita) | I did not notice anything  (kidukimasendeshita) |
| **23** | The older the person was, the more s/he looked dominant. To such extent, they get less attractive. (*oiruhodosihaitekinimie,soredakemiryokutekidenai*) | Only a few faces had neutral expressions  (*Magaodeututteirumonogasukunai*) |
| **24** | I guessed people’s personality based on their impression  (*sono hitono innshoni motoduite sono hitono seikakuwo yosou shimashita*) | I did not notice anything.  (*tokuni kidukimasenn deshita*) |
| **25** | Young woman's face was blotchy  (wakaijyoseinokaogashimidarakedatta) |  |
| **26** | I imagined their expressions in daily life. (*Hudannnohyouzyouhadonnnakannjidearuka*) | I did not notice anything. (*Tokuninanimokidukanakatta*) |
| **27** | Facial expressions and the texture of the skin  (*Hyoujouyahadasitu*) | I did not notice anything.  (*Nanimokidukanakatta*) |
| **28** | I thought what I felt was affected by facial expression, viewing angle, and their clothes.  (hyoujyouyamirukakudo,matakiteiruhukunadoniyottekanjikatagakawarunatoomoimashita) | I guessed the order of the question is controlled by faces.  (kaoniyottetoinojyunbanwosousasiteirunatoomoimashita) |
| **29** | Nothing  (*Tokuninasi*) | I did not notice anything.  (*Tokunikidukazu*) |
| **30** | English written by participant: how were their teeth and eyes is important | English written by participant: there are pictures of same personss different ages |
| **31** | What kind of occupation, what they (*donoyounashokugyounanoka nanioshiteiruhitonanoka*) | I did not notice anything.  (*Tokunikidukanakatta*) |
| **32** | Their actual age (*Zissainonennrei*) | Nothing (*tokuninasi*) |
| **33** | How beauty a face is (*Kaonoutukusisa*) | Contrast, freckle (*konntorasuto,simi*) |
| **34** | Eyes (*Metuki*) | English written by participant: Yes |
| **35** | People who are smiley are credible and attractive.  (*egao no hito wa shinrai dekirushi miryokuteki*)  English written by participant: The person who was smiling looked much more reliable and attractive than ones who were not. | There was one Asian person.  (*Hitori no Asia jin.)*  English written by participant: There was one Asian lady, and others are mostly White. |
| **36** | I thought there were many men with a had a shaved (or bald?) head  (*Bouzugaooitoomoimashita*) | The hairstyle of each man was relatively unique (e.g., a baldhead, white hair). (*Bouzuyasiraganadokamigatokutyoutekinamonogahikakutekiookatta*) |
| **37** | Atmosphere and impressions from eyes (*Hunikitomekaratutawaruinsyou*) | I did not feel strange at all. (*Nanimoiwakanhaarimasendesita*) |
| **38** | How the person is like  (*Donoyounazinnbutuzouka*) | There were no middle-aged people (*Tyunennnohitogainai*) |
| **39** | The number of face lines, the colour of people’s hair (*shiwanokazu,kaminoiro*) | Some faces had many freckles (*Kaogamadaranahitogaita*) |
| **40** | How kind and scared facial expressions are and how many face lines they have  (*hyoujounoyasasisayakowasa,mata,kaonosiwanooosa*) | I did not realise any unusual thing.  (*ijounihakigatukanakatta.*) |
| **41** | Face lines, skin  (*siwa, hada*) | Some people did not show neutral faces.  (*Magaozyanaihitogairu*) |
| **42** | Facial expressions  (Kaonohyoujyou) | Facial expressions were not natural.  (Sizennahyoujyoudehanai) |
| **43** | Whether people are likely to lie and whether I can assign a job to him/her  (*uso wo tsukisouka douka to sigoto wo makaseraresouka douka*) | The colour of photos were processed and it was different from the actual colour.  (*sikisai ga wazato jissaino shashin to kotonaruyouni shiteattakoto*) |
| **44** | If people in a photo are those who are around me, I imagined how I would think and how I would feel.  (*mosi syasinn no hitotati ga jibunn no minomawari no hito nara dou*  *omouka, dou kannjiruka wo kanngaeta*.) | I noticed that some people had many freckles, but I thought it was just intended to be like that. I thought that some people deliberately showed weird facial expressions.  (*kaojyuu ni simi no younamono ga hirogatteiru hito ga itakoto ha kiduitaga,tannnaru seisitu nanoka to omotta. suuninn teido wazato hennna kao wo siteita youna kimo sita.)* |
| **45** | Whether their eyes are warm or not.  (megayasashiikadouka) | There were persons who had many age spots or prominent teeth.  (ijyounisobakasugaooihitoya,ijyounideppanahitogaita) |
| **46** | Atmosphere  (*hunniki*) | No  (*Iie*) |
| **47** | I considered what kind of facial expressions they showed (*Donoyounahyoujouwositeirukawokanngaemashita*) | I thought that how people were taken photos, the amount of light and perspectives (perspective sensation) were different in each person (*Hitoniyotteshashinnnoutsurikatayahikarinoryouyaennkinnkanngakotonarimashita)* |
| **48** | Nothing  (*Tokuninanimo*) | Each background was completely different.  (*Haikeigazennzenntigau*) |
| **49** | Whether the person looked good (kind) or not (*Hitogayosasoukadouka*) | I haven’t noticed anything (*Tokuninanimokidukimasenndeshita*) |
| **50** | I looked at each facial expression carefully. I thought whether they were kind or scary. (*hyoujyouwoyokumimashita.yasashisouka,kowasoukanadowo,kanngaemashita.)* | There were many young and old people. Other than this, I did not notice anything.  (*wakaihitoto,roujinngaooitoomoinashita.soreigaiha,nanimokidukimasenndeshita*.) |
| **51** | It was hard to guess the age of old people. *(nenpainohitononenreihawakarinikui*.) | I did not notice anything.  (*tokunikidukanakatta.)* |
| **52** | Facial expressions, the colour of hair, face lines and backgrounds (Haikwi does not mean anything in Japanese, but it might be “Haikei” = background)  (*hixyoujixyou,kaminoiro,siwa,sonohitonohaikwi*) | I did not notice anything.  (*Nanimokidukimasenndesita*) |
| **53** | How people would show facial expressions in various situations (*Sonohitogairoironajoukyounioitedonnnakaowosuruka*) | All people were foreigners.  (*Subetegaikokujinn*) |
| **54** | Interesting (sometimes translated as: funny)  (Omoshiroi) | No  (*Iie*) |
| **55** | Age and gender  Nennreitoseibetu | I did not realise anything  (*Kiduknakatta*) |
| **56** | Even though people looked old, I thought the actual age would be different from their appearance.  (*kao ga fukete irukara to itte jissai no nenrei ga sou toha kagiranai* *nodewa naidarouka to kanngaeta*) | Nothing  (*toku ni nanimo*) |
| **57** | There are many old people  (*roujin ga ooi*) | It seemed that the colour of a photo was dark and that the experiment would manipulate the impression of each person  (*iroai ga kurakattari insyou wo sousa siteiru youni mieta*) |
| **58** | Atmosphere  (*Hunniki*) | I did not realise anything  (*Kidukanakatta*) |
| **59** | Overall, the gender and age of each person were old or young  *(Seibetuyanennreigadaitaiwakaikatosiwototteiruka*) | I did not know  (*Wakaranakatta*) |
| **60** | I thought that those who were dominant were similar to those who were credible  (*sihaitekinahitoto,sinraidekiruhitoganiteirukigasita*.) | I was impressed by those who were young and had many spots in their face.  (*wakakute,kaonihantengaaruhitogainshountekinikanjitta*) |
